# Termination and Block of Typical Atrial Flutter as Shown by Advisor™ HD Grid Technology

**DOI:** 10.19102/icrm.2021.120118S

**Published:** 2021-01-15

**Authors:** Sri Sundaram, Dan Alyesh, William Choe

**Affiliations:** ^1^South Denver Cardiology Associates, Littleton, CO, USA

**Keywords:** Atrial flutter, arrhythmia, supraventricular tachycardia, ablation

A 65-year-old male patient with no comorbidities presented with palpitations. A diagnosis of typical atrial flutter was established based on a sawtooth pattern on the electrocardiogram with negative flutter waves in the inferior leads and positive flutter waves in V1. The atrial flutter was found to be refractory to medical therapy with β-blockers; thus, the decision to proceed with ablation was made.

In **Figure 1**, the high-density grid catheter (Advisor™ HD Grid Mapping Catheter, Sensor Enabled™) is displayed in a caudal view of the right atrium. Intracardiac electrograms are shown on the bottom. Following the activation sequence of white → red → orange → yellow → green → light blue → dark blue → purple, the counterclockwise mechanism is presented in **Figure 1A**. With an additional ablation lesion at the ablation catheter tip, the tachycardia was terminated and the activation pattern changed from counterclockwise to approaching the cavotricuspid isthmus from a clockwise direction, indicating that block had been achieved; this can be observed with the change in activation pattern in **Figure 1B**. Note that, the earliest color, white, is now seen on the septal side of the cavotricuspid isthmus **(Video 1)**.

The patient remained well and in normal sinus rhythm six weeks after ablation.

## Figures and Tables

**Figure 1:**
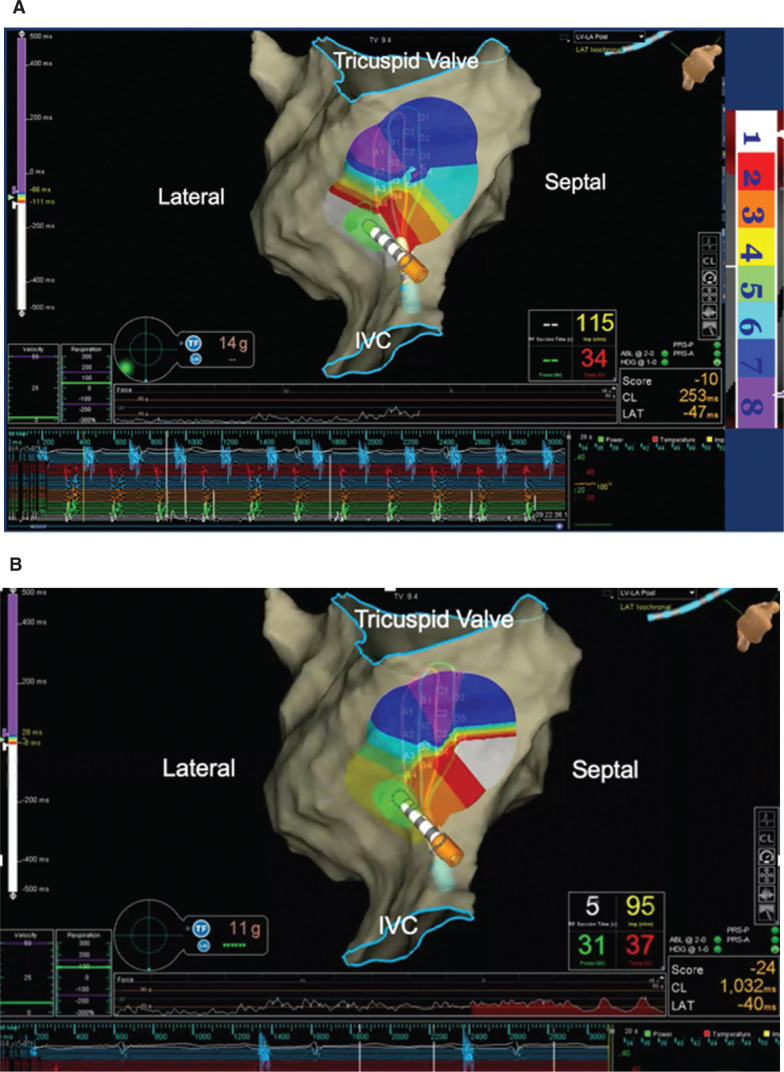
**A:** The Advisor™ HD Grid and ablation catheters are displayed in a caudal view of the right atrium. Intracardiac electrograms are shown on the bottom. Following the activation sequence of white → red → orange → yellow → green → light blue → dark blue → purple, the rhythm was confirmed as typical counterclockwise flutter. **B:** The tachycardia terminated and normal sinus rhythm resumed. Note that the color scheme reversed into a clockwise pattern with this achievement, which indicates that block has been achieved as the activation pattern has changed.

**Video 1.** The cavotricuspid isthmus (CTI) is displayed in a right atrial caudal view. The Advisor™ HD Grid catheter is on the ablation line and reveals the activation sequence to be progressing in a counterclockwise pattern across the CTI with the color scheme of white → red → orange → yellow → green → light blue → dark blue → purple. The electrograms are displayed on the bottom of the video. Initially, the rhythm was typical atrial flutter. However, as the ablation catheter delivered a lesion, the flutter terminated and normal sinus rhythm resumed. With the change in rhythm, the activation pattern on the high-density grid also changed to a clockwise pattern as demonstrated by the change in color scheme.

